# Minority status and readmissions: a moderated mediation model of caregivers’ health literacy

**DOI:** 10.1093/geroni/igaa057.3369

**Published:** 2020-12-16

**Authors:** Nosaiba Rayan-Gharra, Nurit Gur-Yaish, Ksenya Shulyaev

**Affiliations:** University of Haifa, Haifa, Israel

## Abstract

Family caregivers help patients to understand informa¬tion during clinical encounters. Less is known about factors that may affect family caregiver’s Ensuring and Explaining Medical Care (EEMC) during hospitalization and its impact on improved health outcomes. This study examined whether EEMC during hospitalization mediated the association between minority status of patients and 30-day-readmissions, and whether levels of Health Literacy (HL) of caregivers moderated this mediated association. A prospective cohort study of 517 internal medicine patients, Hebrew (general population, coded as 0) and Russian, or Arabic native speakers (minority status, coded as 1), at a tertiary medical center in central Israel, who were accompanied by an informal caregiver. EEMC and HL were patients’ self-reported. 30-day-readmissions were retrieved from the healthcare organization. Logistic regression indicated that minority status was not associated with 30-day readmission when the mediator ICEEMC was not included (B=0.98; p>0.05). However, moderated mediation analysis indicated significant direct (B=-1.08; p=0.003) and indirect effect of minority status on readmission through high ICEEMC during hospitalization among patients who had informal caregivers with high HL level (Mediated effect (ME)=−0.62; CI= -1.07 to -0.29) but not among ones with low HL level (ME= 0.37; CI=-0.24 to 1.06). These findings suggest that caregivers’ high HL may be an essential factor in improving EEMC among minorities. Identifying informal caregivers with high HL level at time of admission to the hospital, and encouraging their involvement during patients’ hospital stay, might be a useful strategy to improve transitions and reducing 30-day readmission, especially among minority patients.

